# Empirical Bayes estimation of posterior probabilities of enrichment: A comparative study of five estimators of the local false discovery rate

**DOI:** 10.1186/1471-2105-14-87

**Published:** 2013-03-06

**Authors:** Zhenyu Yang, Zuojing Li, David R Bickel

**Affiliations:** 1 Ottawa Institute of Systems Biology, Department of Biochemistry, Microbiology, and Immunology, Department of Mathematics and Statistics, University of Ottawa, 451 Smyth Road, Ottawa, Ontario, K1H 8M5 Canada; 2 School of Foundation, Shenyang Pharmaceutical University, No. 103 Wenhua Road, Shenyang, Liaoning, 110016, China

## Abstract

**Background:**

In investigating differentially expressed genes or other selected features, researchers conduct hypothesis tests to determine which biological categories, such as those of the Gene Ontology (GO), are enriched for the selected features. Multiple comparison procedures (MCPs) are commonly used to prevent excessive false positive rates. Traditional MCPs, e.g., the Bonferroni method, go to the opposite extreme: strictly controlling a family-wise error rate, resulting in excessive false negative rates. Researchers generally prefer the more balanced approach of instead controlling the false discovery rate (FDR). However, the q-values that methods of FDR control assign to biological categories tend to be too low to reliably estimate the probability that a biological category is not enriched for the preselected features. Thus, we study an application of the other estimators of that probability, which is called the local FDR (LFDR).

**Results:**

We considered five LFDR estimators for detecting enriched GO terms: a binomial-based estimator (BBE), a maximum likelihood estimator (MLE), a normalized MLE (NMLE), a histogram-based estimator assuming a theoretical null hypothesis (HBE), and a histogram-based estimator assuming an empirical null hypothesis (HBE-EN). Since NMLE depends not only on the data but also on the specified value of *Π*_0_, the proportion of non-enriched GO terms, it is only advantageous when either *Π*_0_ is already known with sufficient accuracy or there are data for only 1 GO term. By contrast, the other estimators work without specifying *Π*_0_ but require data for at least 2 GO terms. Our simulation studies yielded the following summaries of the relative performance of each of those four estimators. HBE and HBE-EN produced larger biases for 2, 4, 8, 32, and 100 GO terms than BBE and MLE. BBE has the lowest bias if *Π*_0_ is 1 and if the number of GO terms is between 2 and 32. The bias of MLE is no worse than that of BBE for 100 GO terms even when the ideal number of components in its underlying mixture model is unknown, but has high bias when the number of GO terms is small compared to the number of estimated parameters. For unknown values of *Π*_0_, BBE has the lowest bias for a small number of GO terms (2-32 GO terms), and MLE has the lowest bias for a medium number of GO terms (100 GO terms).

**Conclusions:**

For enrichment detection, we recommend estimating the LFDR by MLE given at least a medium number of GO terms, by BBE given a small number of GO terms, and by NMLE given either only 1 GO term or precise knowledge of *Π*_0_.

## Background

The development of microarray techniques and high-throughput genomic, proteomic, and bioinformatics scanning approaches (such as microarray gene expression profiling, mass spectrometry, and ChIP-on-chip) has enabled researchers to simultaneously study tens of thousands of biological features (e.g., genes, proteins, single-nucleotide polymorphisms [SNPs], etc.), and to identify a set of features for further investigation. However, there remains the challenge of interpreting these features biologically. For a given set of features, the determination of whether some biological information terms are enriched (i.e., differentially represented), compared to the reference feature set, is termed the *feature enrichment* problem. The biological information term may be, for instance, a Gene Ontology (GO) term [1,2] or a pathway in the Kyoto Encyclopedia of Genes and Genomes (KEGG) [3]. We call this problem the *feature enrichment problem*.

This problem has been addressed using a number of high-throughput enrichment tools, including DAVID, MAPPFinder, Onto-Express and GoMiner [4-7]. Huang et al. [8] reviewed 68 distinct feature enrichment analysis tools. These authors further classified feature enrichment analysis tools into 3 categories: singular enrichment analysis (SEA), gene set enrichment analysis (GSEA), and modular enrichment analysis (MEA). In this article, we propose empirical Bayes solutions to the SEA problem using genes as archetypal features. Without loss of generality, we consider whether some specific biological categories are enriched for differentially expressed genes with respect to the reference genes.

Indeed, like other enrichment-detection methods, our methods apply much more broadly. They can assess enrichment given any sub-list of features selected for future study, not just a list of genes considered differentially expressed. An anonymous referee pointed out these examples of such lists of candidate features that arise in the context of whole genome sequencing: 

•genes with SNPs

•genes with copy number variations

•genes with loss of heterozygosity

These examples and those of our first paragraph do not exhaust contemporary applications, and the feature enrichment problem may occur in unforeseen domains of study. Thus, our illustrative use of differential gene expression as a running example should not be interpreted as a limitation.

Existing enrichment tools mainly address the feature enrichment problem using a p-value obtained from an exact or approximate statistical test (e.g., Fisher’s exact test, the hypergeometric test, binomial test, or the *χ*^2^ test). For each GO term or other biological category, the null hypothesis tested and its alternative hypothesis are as follows:

(1) $$\begin{array}{l} H_{0}:\text{the GO term is not enriched for the preselected genes}\\ H_{1}:\text{the GO term is enriched for the preselected genes} \end{array}$$

Here and in the remainder of the paper, we use GO terms as concrete examples of biological categories without excluding applications of the methods to categories from other relevant databases. The general process begins as follows: 

•For each GO term, construct Table 1 based on the preselected genes (e.g., differentially expressed (DE) genes) and reference genes (e.g., all genes measured in a microarray experiment).

•Compute the p-value for each GO term using a statistical test that can detect enrichment for the preselected genes.

**Table 1:** The number of differentially expressed (DE) and equivalently expressed (EE) genes in a GO category

	**DE genes**	**EE gene**	**Total**
In GO category	*x*_1_	*x*_2_	*x*_1_+*x*_2_
Not in GO category	*n* − *x*_1_	*N* − *n* − *x*_2_	*N* − *x*_1_ − *x*_2_
Total	*n*	*N* − *n*	*N*

Here, *x*_*i*_ (*i*=1,2) is the number of DE (*i*=1) or EE genes (*i*=2) in the GO category; *n* is the total number of DE genes; *N* is the total number of reference genes.

Multiple comparison procedures (MCPs) are then applied to the resulting p-values to prevent excessive false positive rates. The false discovery rate (FDR) [9] is frequently used to control the expected proportion of incorrectly rejected null hypotheses in gene enrichment studies [10-12] because it has lower false negative rates than Bonferroni correction and other methods of controlling the family-wise error rate. Methods of FDR control assign q-values [13] to biological categories, but q-values are too low to reliably estimate the probability that the biological category is not enriched for the preselected features. Thus, we study application of better estimators of that probability, which is technically known as the local FDR (LFDR). Hong et al. [14] used an LFDR estimator to solve a GSEA problem and pointed out that this was less biased than the q-value for estimating the LFDR, the posterior probability that the null hypothesis is true.

Efron [15,16] devised reliable LFDR estimators for a range of applications in microarray gene expression analysis and other problems of large-scale inference. However, whereas microarray gene expression analysis takes into account tens of thousands of genes, the feature enrichment problem typically concerns a much smaller number of GO terms. While these methods are appropriate for microarray-scale inference, they are less reliable for enrichment-scale inference [17-19]. Thus, we will specifically adapt LFDR estimators that are appropriate for smaller-scale inference to address the SEA problem. Again, we will focus on genes and GO terms for the sake of concreteness. Nevertheless, the estimators used can be applied to other features and to other biological terms (e.g., metabolic pathways).

The sections of this paper are arranged as follows. We first introduce some preliminary concepts in the feature enrichment problem. Next, two previous LFDR estimators and three new LFDR estimators are described. Following this, we compare the LFDR estimators by means of a simulation study and an application to breast cancer data. Finally, we draw conclusions and make recommendations on the basis of our results.

## Preliminary concepts

The feature enrichment problem described in the Background section is stated here more formally for the application of LFDR methods in the next section.

### Likelihood functions

In Table 1, *x*_1_ and *x*_2_ are the observed numbers DE genes and EE genes in a given GO category, respectively. Whereas *n* is the total number of DE genes, *N* is the total number of reference genes. Thus, *N* − *n* is the total number of EE genes. The columns gives the numbers of DE genes and EE genes, and the rows give the numbers of genes in the GO category and outside the GO category.

Let *x*_1_ and *x*_2_, respectively, denote the random numbers of DE and EE genes in a GO category. The observed values *x*_1_ and *x*_2_ are modeled as realizations of *x*_1_ and *x*_2_. *x*_1_ and *x*_2_ follow binomial distributions, namely, *X*_1_ ∼ Binomial(*n*,* π *_1_) and *X*_2_ ∼ Binomial(*N* − *n*,*π*_2_), where *π*_1_ and *π*_2_ are the probabilities that a gene is DE and EE, respectively, given that it is in the GO category. Under the assumption that *x*_1_ and *x*_2_ are independent, the *unconditional likelihood* is

(2) L(π1,π2;x1,x2,n,N)=Pr(X1=x1,X2=x2;π1,π2,n,N)=nx1N−nx2π1x1(1−π1)n−x1π2x2(1−π2)N−n−x2,

where 0 ≤ *x*_1_ ≤ *n*, 0 ≤ *x*_2_ ≤ *N* − *n*, and 0 ≤ *π*_*i*_ ≤ 1, *i*=1,2.

If we define

(3) $$\lambda =\ln[\pi_{2}/(1-\pi_{2})],$$

and

(4) $$\theta =\ln[\pi_{1}/(1-\pi_{1})]-\lambda$$

then *θ* is the parameter of interest, representing the *log odds ratio* of the GO term, and *λ* is a nuisance parameter. Under the new parametrization, the unconditional likelihood function (2) is

(5) L(θ,λ;x1,x2,n,N)=nx1N−nx2×ex1(θ+λ)ex2λ(1+eθ+λ)n(1+eλ)N−n,

where 0 ≤ *x*_1_ ≤ *n* and 0 ≤ *x*_2_ ≤ *N* − *n*.

In equation (5), we take the interest parameter *θ* and also the nuisance parameter *λ* into consideration. Consider statistics *T* and *S*, functions of *x*_1_ and *x*_2_, such that *T*(*X*_1_,*X*_2_) = *X*_1_ and *S*(*X*_1_,*X*_2_) = *X*_1_+*X*_2_. Thus, *T* represents the number of DE genes in a GO category, and *S* represents the number of total genes in a GO category. Let *t* and *s* be the observed values of *T* and *S*. The probability mass function of *T*(*x*_1_,*x*_2_) = *t* evaluated at *S*(*x*_1_,*x*_2_) = *x*_1_+*x*_2_ = *s*, say Pr(*T* = *t*|*S* = *s*;*θ*,*λ*,*N*,*n*), does not depend on the nuisance parameter *λ*[19]. See also Example 8.47 of Severini[20]. Thus, we derive the conditional probability mass function

(6) fθ(t|s)=Pr(T=t|S=s;θ,n,N)=ntN−ns−tetθ∑j=max(0,s+n−N)min(s,n)njN−ns−jejθ

understood as a function of *t*.

By eliminating the nuisance parameter *λ*, we can reduce the original data *x*_1_ and *x*_2_ by considering the statistic *T* = *t*. However, the use of the conditional probability mass function requires some justification because of concerns about losing information during the conditioning process. Unfortunately, in the presence of the nuisance parameter, the statistic *S*(*X*_1_,*X*_2_) = *X*_1_+*X*_2_ is not an ancillary statistic for the parameter of interest. In other words, the probability mass function of the conditional variable *S*(*X*_1_,*X*_2_) may contain some information about parameter *θ*[20]. However, following the explanation of Barndor-Nielsen and Cox ([21], §2.5), the expectation value of statistic *S*(*X*_1_,*X*_2_) equals the nuisance parameter. Hence, from the observation of *S*(*X*_1_,*X*_2_) alone, the distribution of *S*(*X*_1_,*X*_2_) contains little information about *θ*[21]. *S*(*X*_1_,*X*_2_) satisfies the other 3 conditions of an ancillary statistic defined by Barndor-Nielsen and Cox [21]: parameters *θ* and *λ* are variation independent; (*T*(*X*_1_,*X*_2_),*S*(*X*_1_,*X*_2_)) is the minimal sufficient statistic; and the distribution of *T*(*X*_1_,*X*_2_), given *S*(*X*_1_,*X*_2_) = *s*, is independent of the parameter of interest, *θ*, given the nuisance parameter *λ*. Therefore, the probability mass function of *S*(*X*_1_,*X*_2_) contains little information about the value of *θ*.

### Hypotheses and LFDRs

Considering GO term *i*, we denote the *T*, *S*, *t*, *s*, and *θ* used in equation (6) as *T*_*i*_, *S*_*i*_, *T*_*i*_, *S*_*i*_, and *θ*_*i*_. From Table 1, hypothesis comparison (1) of GO term *i* is equivalent to

(7) $$H_{0}:\theta_{i}=0\text{versus}H_{1}:\theta \neq 0.$$

Let **S** = 〈*S*_1_,*S*_2_,⋯,*S*_*m*_〉 and **S** = 〈*S*_1_,*S*_2_,⋯,*S*_*m*_〉. Let BF_*i*_ denote the *Bayes factor*of GO term *i*:

(8) $${\text{BF}}_{i}=\frac{\text{Pr}(T_{i}=t_{i}|S=s,\theta_{i}\neq 0)}{\text{Pr}(T_{i}=t_{i}|S=s,\theta_{i}=0)}.$$

It is called the Bayes factor because it yields posterior odds when multiplied by prior odds. More precisely, the *posterior odds* of the alternative hypothesis corresponding to GO term *i* is

(9) $$\omega_{i}=\frac{\text{Pr}(\theta_{i}\neq 0|t_{i})}{\text{Pr}(\theta_{i}=0|t_{i})}={\text{BF}}_{i}\times \frac{\left(1-\Pi_{0}\right)}{\Pi_{0}},$$

where *Π*_0_ is the *prior conditional probability* that a GO term is not enriched for the preselected genes given **s**, i.e., *Π*_0_ = Pr(*θ*_*i*_ = 0|**S** = **s**). Thus, (1 − *Π*_0_)/*Π*_0_ is the *prior odds* of the alternative hypothesis of enrichment. According to Bayes’ theorem, the LFDR of GO term *i* is

(10) $${\text{LFDR}}_{i}=\text{Pr}(\theta_{i}=0|t_{i})=\frac{1}{1+\omega_{i}},$$

where *ω*_*i*_ is defined in equation (9).

## Methods

This section is divided into two parts: 

1. **Previous LFDR estimators.** While not unique to this paper, these methods are included for comparison.

2. **New LFDR estimators.** Our main methodological innovations are the uses of a conditional probability mass function and of normalized maximum likelihood for LFDR estimation.

The other original contributions of this paper are the estimator comparisons of the next section. The comparisons are made by simulation and by a case study.

### Previous LFDR estimators

#### Binomial-based LFDR estimator

The version of the FDR that generalizes the LFDR is the *nonlocal FDR*, which is defined as the ratio of the expected number of false discoveries to the expected total number of discoveries [17]. In our running example, a *discoveryof enrichment*is a rejection of the null hypothesis of non-enrichment at some significance level *α*, and a *false discoveryof enrichment* is a discovery of enrichment corresponding to a case of no actual enrichment. (This FDR has been called the “Bayesian FDR” [22] to distinguish it from the FDR of Benjamini and Hochberg [9]).

Let *α* denote any significance level chosen to be between 0 and 1. For all GO terms of interest, the nonlocal FDR may be estimated by

(11) $$\begin{array}{ll} \hat{\text{FDR}}(\alpha ) & =\min\left(\frac{\mathrm{m\alpha}}{\overset{m}{\underset{j=1}{\sum }}1_{\left\{p_{j}\leq \alpha \right\}}},1\right),\; \end{array}$$

where *m* is the number of GO terms, *p*_*j*_ is the p-value of GO term *j*, and $1_{\left\{p_{j}\leq \alpha \right\}}$ is the indicator such that $1_{\left\{p_{j}\leq \alpha \right\}}=1$ if *p*_*j*_ ≤ *α* is true and $1_{\left\{p_{j}\leq \alpha \right\}}=0$ otherwise. Thus, $\overset{m}{\underset{j=1}{\sum }}1_{\left\{p_{j}\leq \alpha \right\}}$ represents the number of discoveries of enriched GO terms, and *mα* estimates the number of such discoveries that are false.

Let *r*_*i*_ be the rank of the p-value of GO term *i*, e.g., *r*_*i*_ = 1 if the p-value of GO term *i* is the smallest among all p-values of *m* GO terms. Based on a modification of equation (11), the *binomial-based estimator*(BBE) of LFDR of the GO term *i* is

(12) $${\hat{\text{LFDR}}}_{i}=\left\{\begin{array}{ll} \min\left(\frac{mp_{2r_{i}}}{2r_{i}},1\right), & r_{i}\leq \frac{m}{2},\\ 1, & r_{i}>\frac{m}{2}. \end{array}\right)$$

It is conservative in the sense that it tends to overestimate LFDR [17].

#### Histogram-based LFDR estimator

Efron [15,16] devised reliable histogram-based LFDR estimators for a range of applications in microarray gene expression analysis and other problems of large-scale inference. Let *z*_*i*_ = *Φ*^ − 1^(*p*_*i*_) be the z-transformed statistic of GO term *i*, where *Φ* is the standard normal cumulative distribution function (cdf) and *p*_*i*_ is the 2-sided p-value of GO term *i*. For each GO term, the density is a mixture of the form

(13) $$f(z_{i})=\Pi_{0}f_{0}(z_{i})+(1-\Pi_{0})f_{1}(z_{i}),$$

where *f*_0_ is the density function of *z* for the non-enriched GO terms, *f*_1_ is that for the enriched GO terms, and *Π*_0_ is the probability that a GO term is non-enriched. The histogram-based LFDR of GO term *i* is estimated by equation (14):

(14) $${\hat{\text{LFDR}}}_{i}=\frac{\hat{f_{0}}(z_{i})}{\hat{f}(z_{i})},$$

where $\hat{f}$ is the estimator of *f* that is estimated by a nonparametric Poisson regression method [15,16]. We call ${\hat{\text{LFDR}}}_{i}$*the histogram-based estimator* (HBE) if the density function *f*_0_ is assumed to be standard normal, *N*(0,1), and *the histogram-based estimator with empirical null* (HBE-EN) if the density function *f*_0_ is estimated based on the truncated maximum likelihood technique of [16]. Dalmasso et al. [23] compared the precursor of HBE-EN [15] to other LFDR estimators.

### New LFDR estimators

#### Type II maximum likelihood estimator

Bickel [17] follows Good [24] in calling the maximization of likelihood over a hyperparameter *Type II maximum likelihood* to distinguish it from the usual *Type I maximum likelihood*, which pertains only to models that lack random parameters. Type II maximum likelihood has been applied to parametric mixture models (PMMs) for the analysis of microarray data [25,26], proteomics data [18], and genetic association data [27]. In this section, we adapt the approach to the feature enrichment problem by using the conditional probability mass function defined above. The particular models we use in this framework correspond to new methods of enrichment analysis.

Let $G(s)=\{g_{\theta }(∙|s);\theta \geq 0\}$ be a parametric family of probability mass functions with

(15) $$\begin{array}{ll} g_{\theta }(∙|s) & =\frac{1}{2}\times \left[f_{\theta }(∙|s)+f_{-\theta }(∙|s)\right],\; \end{array}$$

where *f*_*θ*_(∙|**s**) is defined in equation (6). We define the *k-component PMM*as

(16) $$\begin{array}{ll} g(∙|s;\theta_{0},\ldots ,\theta_{k-1},\Pi_{0},\ldots ,\Pi_{k-1}) & =\overset{k-1}{\underset{j=0}{\sum }}\Pi_{j}g_{\theta_{j}}(∙|s),\; \end{array}$$

where *θ*_0_ = 0 and *θ*_*j*_≠*θ*_*J*_ for all $j,J\in \left\{0,\ldots ,k-1\right\}$ such that *j*≠*J*.

Let **T** = 〈*T*_1_,*T*_2_,⋯,*T*_*m*_〉 and **T** = 〈*T*_1_,*T*_2_,⋯,*T*_*m*_〉 be vectors of the *T*_*i*_s and *T*_*i*_s used in equation (8). Assuming *T*_*i*_ is independent of *T*_*j*_ and *S*_*j*_ for all $i,j\in \left\{1,\ldots ,m\right\}$ such that *j*≠*J*. *i*≠*j*, the joint probability mass function is

(17) $$\begin{array}{lll} g & (t|s;\theta_{0},\ldots ,\theta_{k-1},\Pi_{0},\ldots ,\Pi_{k-1})\; & \;\\ & =\overset{m}{\underset{i=1}{\prod }}g(t_{i}|s;\theta_{0},\ldots ,\theta_{k-1},\Pi_{0},\ldots ,\Pi_{k-1})\;\\ & =\overset{m}{\underset{i=1}{\prod }}g(t_{i}|s_{i};\theta_{0},\ldots ,\theta_{k-1},\Pi_{0},\ldots ,\Pi_{k-1}),\; & \; \end{array}$$

where *S*_*i*_ is the observed value of *S*_*i*_ for GO term *i*, and **S**=〈*S*_1_,*S*_2_,⋯,*S*_*m*_〉.

Moreover, we assume that for a given number of genes in GO term *i*, *T*_*i*_(*i* = 1,…,*m*) satisfies the *k*-component PMM shown in equation (16). In other words, we assume that the possible log odds ratios of GO term *i* are the *θ*_0_,*θ*_1_,*θ*_2_,…,*θ*_*k* − 1_ of equation (16) if the alternative hypothesis *H*_1_ in hypothesis comparison (7) is true.

Therefore, the log-likelihood function under the *k*-component PMM for all GO terms is

(18) $$\begin{array}{lll} \log & L(\theta_{0},\ldots ,\theta_{k-1},\Pi_{0},\ldots ,\Pi_{k-1})\; & \;\\ & =\log g(t|s;\theta_{0},\ldots ,\theta_{k-1},\Pi_{0},\ldots ,\Pi_{k-1})\; & \;\\ & \text{=}\overset{m}{\underset{i=1}{\sum }}\left[\log\overset{k-1}{\underset{j=0}{\sum }}\Pi_{j}g_{\theta_{j}}(t_{i}|s_{i})\right].\; \end{array}$$

The LFDR of GO term *i* is estimated by

(19) $${\hat{\text{LFDR}}}_{i}^{\left(k\right)}=\frac{{\hat{\Pi }}_{0}g_{\theta_{0}}(t_{i}|s_{i})}{g(t_{i}|s_{i};\theta_{0},{\hat{\theta }}_{1},\ldots ,{\hat{\theta }}_{k-1},{\hat{\Pi }}_{0},\ldots ,{\hat{\Pi }}_{k-1})},$$

where ${\hat{\theta }}_{1},\ldots ,{\hat{\theta }}_{k-1}$ and ${\hat{\Pi }}_{0},\ldots ,{\hat{\Pi }}_{k-1}$ are maximum likelihood estimates of *θ*_1_,…,*θ*_*k* − 1_ and *Π*_0_,…,*Π*_*k* − 1_ in equation (18). We call ${\hat{\text{LFDR}}}_{i}^{\left(k\right)}$ the *k-component maximum likelihood estimator* (MLE*k*). Our LFDRenrich and LFDRhat software suites of R functions that implement MLE2 and MLE3 are now available at http://www.statomics.com. Moreover, ${\hat{\theta }}_{i}$ (*i* = 1,…,*k* − 1;*k* = 2,3) and ${\hat{\Pi }}_{j}$ (*j* = 0,…,*k* − 1;*k* = 2,3), also in LFDRenrich and LFDRhat, are maximum likelihood estimators of *θ*_*i*_ (*i* = 1,…,*k* − 1;*k* = 2,3) and *Π*_*j*_ (*j* = 0,…,*k* − 1;*k* = 2,3) under given constraints.

#### LFDR estimator based on the normalized maximum likelihood

Combining equations (9)-(10), we obtain

(20) $${\text{LFDR}}_{i}={\left(1+{\text{BF}}_{i}\times \frac{\left(1-\Pi_{0}\right)}{\Pi_{0}}\right)}^{-1}.$$

Therefore, given a guessed value of *Π*_0_, we may use an estimator of the Bayes factor to estimate the LFDR of a GO term.

We now develop such an estimator of the Bayes factor. For GO category *i*, let $E_{i}$ stand for the set of all probability mass functions defined on {0,1,…,*s*_*i*_}, the set of all possible values of *T*_*i*_. Based on hypothesis comparison (7), the set of log odds ratios, denoted as ℋ, is {0} under the null hypothesis and is $R\setminus \left\{0\right\}=\left\{\theta \in R:\theta \neq 0\right\}$, the set of all real values except 0, under the alternative hypothesis. With the assumption that random variable *T*_*i*_ is independent of random variable *S*_*j*_ for any *i*≠*j*, the *regret* of a predictive mass function $\bar{f}\in E_{i}$ is a measure of how well it predicts the observed value *t*_*i*_∈{0,1,…,*s*_*i*_}. The regret is defined as

(21) $$\text{reg}(\bar{f},t_{i}|s_{i};ℋ)=\log\frac{f_{{\hat{\theta }}_{i}(t_{i}|s_{i})}(t_{i}|s_{i})}{\bar{f}(t_{i}|s_{i})},$$

where ${\hat{\theta }}_{i}(t_{i}|s_{i})$ is a Type I MLE with respect to ℋ under observed values *T*_*i*_ given *S*_*i*_[28,29].

For all members of $E_{i}$, the *optimal predictive conditional probability mass function* of GO category *i* and the hypothesis that $\theta_{i}\in ℋ$ is denoted by $f_{i}^{†}(∙|s_{i};ℋ)$. It minimizes the maximal regret in sample space {0,1,…,*s*_*i*_} in the sense that it satisfies

(22) $$f_{i}^{†}(∙|s_{i};ℋ)=\arg\underset{\bar{f}\in E_{i}}{\min}\underset{t\in \left\{0,1,\ldots ,s_{i}\right\}}{\max}\text{reg}(\bar{f},t|s_{i};ℋ).$$

It is well known [28] that the predictive probability mass function that satisfies equation (22) is

(23) $$\begin{array}{ll} f_{i}^{†}(t_{i}|s_{i};ℋ) & =\frac{\underset{\theta \in ℋ}{\max}f_{\theta }(t_{i}|s_{i})}{K_{i}^{†}(ℋ)},\; \end{array}$$

where *f*_*θ*_(*t*_*i*_|*s*_*i*_) is the conditional probability mass function defined in equation (6), and $K_{i}^{†}(ℋ)$ is a constant defined as

(24) Ki†(ℋ)=maxθ∈ℋfθ(y|si)=∑y=max(0,si+n−N)min(si,n)maxθ∈ℋfθ(y|si)=∑y=max(0,si+n−N)min(si,n)nyN−nsi−yeyθ^i(y)∑j=max(0,si+n−N)min(si,n)njN−nsi−jejθ^i(y),

where

(25) $${\hat{\theta }}_{i}(y)=\arg\underset{\theta \in ℋ}{\max}f_{\theta }(y|s_{i}).$$

We call $f_{i}^{†}(t_{i}|s_{i};ℋ)$ the *normalized maximum likelihood* (NML) associated with the hypothesis that $\theta_{i}\in ℋ$.

Thus, BF_*i*_ is approximated by

(26) $${\hat{\text{BF}}}_{i}^{†}=\frac{f_{i}^{†}(t_{i}|s_{i};R\setminus \left\{0\right\})}{f_{i}^{†}(t_{i}|s_{i};0)},$$

which we call the *NML ratio*. (More generally, the logarithm of an NML ratio is interpreted as a measure of the evidential support for the alternative hypothesis over the null hypothesis [29,30]). Therefore, by combining equations (8) and (9), if we guess the prior probability *Π*_0_, the LFDR estimate of GO category *i* in the hypothesis comparison (7) is

(27) $${\hat{\text{LFDR}}}_{i}^{†}={\left[1+\frac{1-\Pi_{0}}{\Pi_{0}}\times {\hat{\text{BF}}}_{i}^{†}\right]}^{-1},$$

where ${\hat{\text{BF}}}_{i}^{†}$ is defined in equation (26). We call this LFDR estimator the *NML estimator* (NMLE).

To assess the reliability of NML ratio ${\hat{\text{BF}}}_{i}^{†}$ for a particular data set, it will be compared to an empirical Bayes estimate of the Bayes factor that unlike NML, simultaneously takes all GO terms into account. Equations (19) and (20) suggest

(28) $${\hat{\text{BF}}}_{i}=\frac{1-{\hat{\text{LFDR}}}_{i}^{\left(k\right)}}{{\hat{\text{LFDR}}}_{i}^{\left(k\right)}}\times \frac{1-{\hat{\Pi }}_{0}}{{\hat{\Pi }}_{0}}$$

as the empirical Bayes estimator of BF_*i*_.

## Results and discussion

In this section, we compared the LFDR estimators using simulation data and breast cancer data.

For each GO category, the p-value used in BBE to estimate LFDR is computed based on the 2-sided Fisher’s exact test. In computing MLE*k* (*k* = 2,3), *θ*_*i*_ (*i* = 1,…,*k* − 1) in equation (18) was constrained to lie between 0 and 10, whereas *Π*_*i*_ (*i* = 0,…,*k* − 1) in equation (18) was allowed to take any value between 0 and 1 such that $\overset{k-1}{\underset{i=0}{\sum }}\Pi_{i}=1$.

### Simulation studies

The aim of the following simulation studies is to compare the LFDR estimation biases of BBE, MLE2, MLE3, HBE, and HBE-EN. NMLE is not taken into account because its performance depends not only on the data, but also on the specified prior probability *Π*_0_.

The simulation setting involves 10,000 genes in a microarray with 200 genes identified as DE and 100 GO terms. We conducted a separate simulation study using each of these values of *Π*_0_: 50%, 60%, 70%, 80%, 90%, and 94%.

Since the PMM behind MLE is optimal when the number of enriched GO terms is equal to the number non-enriched GO terms, we assessed the sensitivity of MLE to that symmetry assumption by using strongly asymmetric log odds ratios and by using symmetric ones. For each GO term, two configurations were used in this simulation to choose log odds ratios: the *asymmetric configuration* shown in equation (29) and the *symmetric configuration* shown in equation (30). We used these values of odds ratio of the *i*th GO term:

(29) $$\phi_{i}^{\text{asymmetric}}=\left\{\begin{array}{ll} \frac{5i}{100(1-\Pi_{0})}, & 1\leq i\leq 100(1-\Pi_{0}),\\ 0, & 100(1-\Pi_{0})<i\leq 100; \end{array}\right)$$

(30) $$\phi_{i}^{\text{symmetric}}=\left\{\begin{array}{ll} \frac{5\times 2i}{100(1-\Pi_{0})}, & 1\leq i\leq 50(1-\Pi_{0}),\\ 5-\frac{5\times 2i}{100(1-\Pi_{0})}, & 50(1-\Pi_{0})<i\leq 100(1-\Pi_{0}),\\ 0, & 100(1-\Pi_{0})<i\leq 100. \end{array}\right)$$

Considering the log odds ratios of all GO terms in each simulation study, we generated Table 1 for GO term *i* and for each of the 20 simulated data sets as follows: 

•*x*_1_ is generated from a binomial distribution with parameter *π*_1_ used in equation (2); *π*_1_ is a real value randomly picked from 0 to 1.

•*x*_2_ is obtained from a binomial distribution with parameter $\pi_{2}={\left[\frac{(1-\pi_{1})\times 2^{\phi_{i}}}{\pi_{1}}+1\right]}^{-1}$, obtained by solving

(31) $$\phi_{i}=\underset{2}{\log}[\pi_{1}/(1-\pi_{1})]-\underset{2}{\log}[\pi_{2}/(1-\pi_{2})].$$

Thus, according to equation (4), we obtain *ϕ*_*i*_ = *θ*_*i*_ log2*e* for GO term *i*. Each of those artificial data sets represents what might have been a real data set such as that of the next subsection.

The p-value of each GO term used in BBE, HBE, and HBE-EN is obtained from the 2-sided Fisher’s exact test. The *k*-component PMM (*k* = 2 or *k* = 3) used in MLE is shown in equation (16) with $\Pi_{j}=\left(1-\Pi_{0}\right)/k\;\left[j=1,\ldots ,k\right]$ and $g_{\theta_{i}}(t_{i}|s_{i})$ defined in equation (15). For every log odds ratio sequence, we estimated the LFDR for each GO term and each data set using BBE, MLE2, MLE3, HBE, and HBE-EN. We compared the performances of the 5 estimators by means of computing the absolute value of the estimated LFDR bias. The true LFDR is computed by equation (10), where

f0(ti)=ntiN−nsi−ti∑j=max(0,si+n−N)min(si,n)njN−nsi−j

and *f*_1_(*t*_*i*_) is computed by

$$\frac{1}{J}\overset{J}{\underset{j=1}{\sum }}f_{\theta_{j}}(t_{i}|s_{i}),$$

where *f*_*θ*_(*t*|*s*) is defined in equation (6).

**Figure 1:**
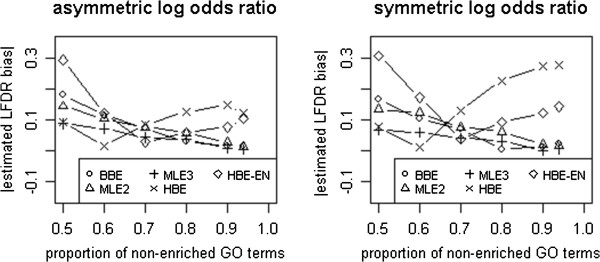
The performance of LFDR estimators for 100 GO terms with asymmetric and symmetric log odds ratios.

Figure 1 shows the performance comparisons of the 5 LFDR estimators (i.e., BBE, MLE2, MLE3, HBE, and HBE-EN) for simulation data obtained from asymmetric and symmetric log odds ratios. The absolute LFDR biases estimated by BBE, MLE2, MLE3, and HBE-EN are similar. The absolute bias of LFDR estimated by HBE on symmetric log odds ratios is a little higher than that on asymmetric log odds ratios when the proportion of non-enriched GO terms is greater than 80%. Therefore, the estimated LFDR biases of the estimators are not strongly affected by whether the log odds ratios are symmetric or asymmetric.

**Figure 2:**
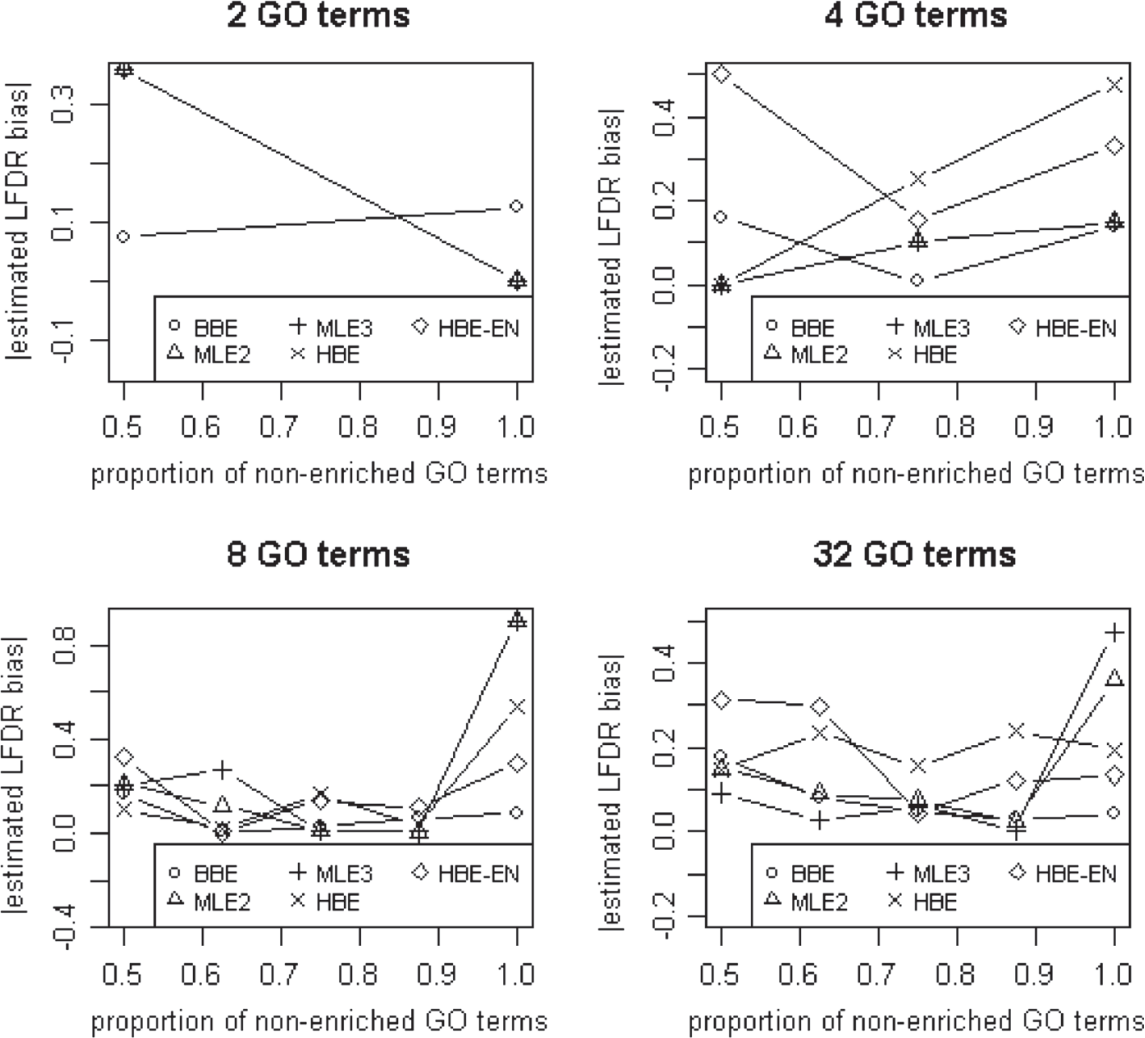
The performance of LFDR estimators for 2, 4, 8, and 32 GO terms with log odds ratios.

To assess the performance of the 5 estimators for smaller GO terms, we added simulation studies using 2, 4, 8, and 32 as the total number of GO terms. The proportion of non-enriched GO terms (*Π*_0_) and log odds ratios of simulation studies are shown in Table 2. The simulation studies were otherwise the same as those for 100 GO terms. Figure 2 shows the performance of LFDR estimators by means of computing the absolute estimated LFDR bias for 2, 4, 8, and 32 GO terms with log odds ratios based on formulas shown in Table 2.

**Table 2:** The proportion of non-enriched GO terms and the log2 odds ratios of GO terms used in the simulation studies

**Number of GO terms**	***Π***_**0**_	**log2 odds ratio (*ϕ***_***i***_**)**	
		0.5,	*i* = 1
2	50.0%	0,	*i* = 2
	100.0%	0,	*i* = 1,2
		3,	*i* = 1
	50.0%	−3,	*i* = 2
		0,	*i* = 3,4
		0.5,	*i* = 1
4	75.0%	0,	*i* = 2,…,4
	100.0%	0,	*i* = 1,…,4
		(0.5 +1.5×(*i* − 1)),	*i*=1,2
	50.0%	−(0.5 +1.5×(*i* − 3)),	*i* = 3,4
		0,	*i* = 5,…,8
		(0.5 +1.5×(*i* − 1)),	*i* = 1,2
	62.5%	−(0.5 +1.5×(*i* − 2)),	*i* = 3
		0,	*i* = 4,…,8
		2,	*i* = 1
8	75.0%	−2,	*i* = 2
		0,	*i*=3,…,8
		2,	*i* = 1
	87.5%	0,	*i* = 2,…,8
	100.0%	0,	*i* = 1,…,8
		(0.32 +0.64×(*i* − 1)),	*i* = 1,…,8
	50.0%	−(0.32 +0.64×(*i* − 9)),	*i* = 9,…,16
		0,	*i* = 17,…,32
		(0.8 +0.8×(*i* − 1)),	*i* = 1,…,3
	62.5%	−(0.8 +0.8×(*i* − 4)),	*i* = 4,…,6
		0,	*i* = 7,…,32
		(0.2 +1.6×(*i* − 1)),	*i* = 1,2
32	75.0%	−(0.2 +1.6×(*i* − 1)),	*i* = 3,4
		0,	*i* = 5,…,32
		1.8,	*i* = 1
	87.5%	−1.8,	*i* = 2
		0,	*i* = 3,…,32
	100.0%	0,	*i* = 1,…,32

Here, index *i* labels the GO term.

**Figure 3:**
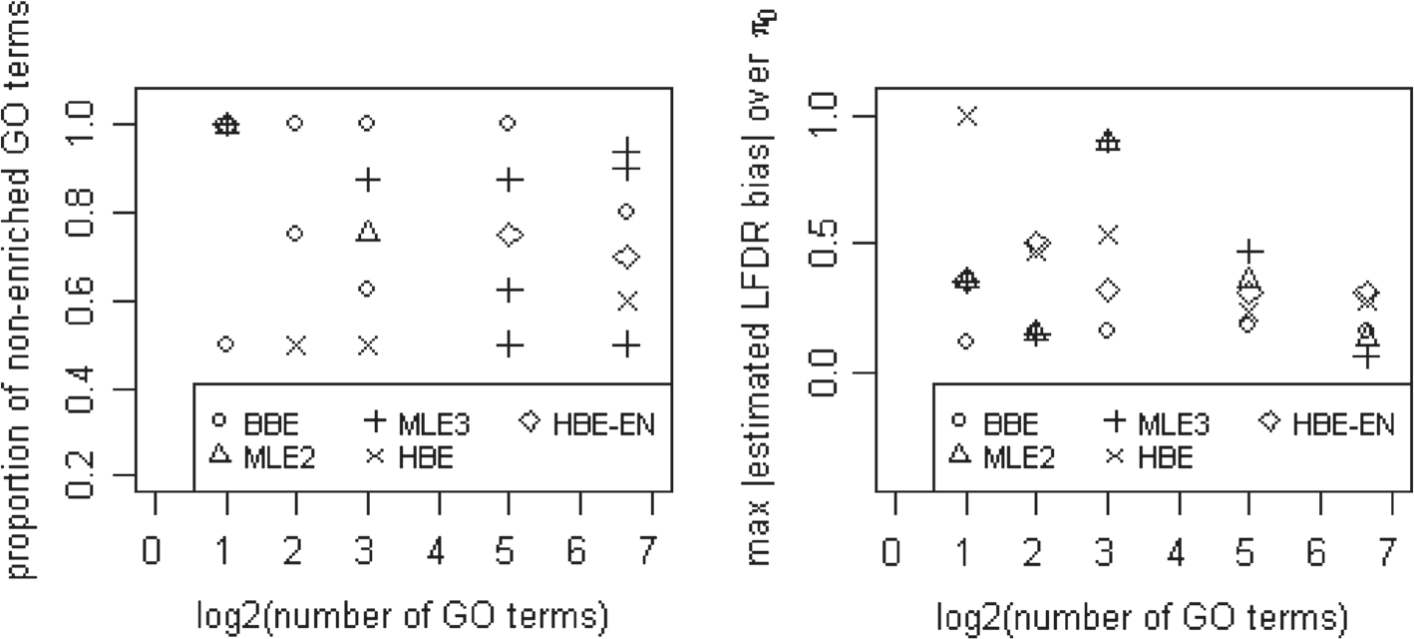
**The performance comparison of LFDR estimators based on which estimator achieves the lowest absolute estimated LFDR bias at each combination of*Π***_**0**_** and *m * (left) and, for each estimator, the maximum of absolute estimated LFDR bias over the proportion of non-enriched GO terms at each value of *m * (right).**

Considering every (*m*,*Π*_0_) pair of the simulation studies with symmetric log odds ratios for the case of 100 GO terms, we recorded the LFDR estimator with the lowest absolute estimated LFDR bias among the 5 LFDR estimators (BBE, MLE2, MLE3, HBE, and HBE-EN). Moreover, we determined the maximum absolute LFDR bias over the proportion of non-enriched GO terms (*Π*_0_) in order to evaluate the worst-case bias of each estimator at each value of *m*. Figure 3 shows the results.

### Breast cancer data analysis

The single-channel microarray data set used here to illustrate our new methods is from an experiment applying an estrogen treatment to cells of a human breast cancer cell line [31]. The Affymetrix human genome U-95Av2 genechip data are from four samples from an estrogen receptor positive breast cancer cell line. Two of the samples were exposed to estrogen and then harvested after 10 hours. The remaining two samples were left untreated and then harvested after 10 hours. For simplicity of terminology, we call probes in the microarray experiment “genes.” The relevant data consist of measurements of gene expression across the reference class of 12,625 genes. The purpose of the study was to determine which genes are affected by the estrogen treatment. (For further information concerning the data, see Gentleman et al. [32].)

We applied the R function expresso in the affy package [33] of Bioconductor [34] to convert the raw probe intensities from the the CEL data files to logarithms of gene expression levels without background correction. In doing so, we applied the “quantiles,” “pmonly,” and “medianpolish” [35] preprocessing settings.

We selected as genes of interest those that were differentially expressed between the treatment group and the control group according to the following criterion. Using the LFDR as the probability that a gene is EE, we considered genes with LFDR estimates below 0.2 as DE. In other words, we selected as DE genes those that were differentially expressed with estimated posterior probability of at least 80%. Considering four samples of each gene in the microarray, we used the unpaired t-test with equal variances to compute the p-value. The LFDR of every gene is estimated using the theoretical null hypothesis method of Efron [15,16]; the empirical null hypotheses method can lead to excessive bias due to deviations from normality [36]. When we compared gene expression data for the presence and absence of estrogen after 10 hours of exposure, we obtained 74 DE genes.

**Figure 4:**
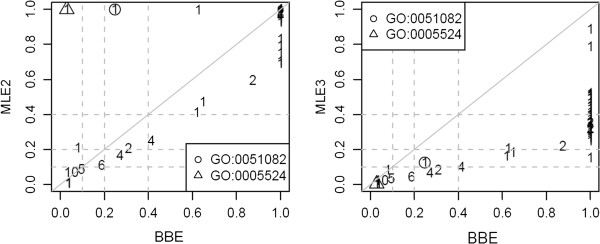
**Comparison of the LFDR estimated by BBE with the LFDR estimated by MLE2 (left) and MLE3 (right).** Each integer represents a number of GO terms. Intergers > 1 indicate ties.

**Figure 5:**
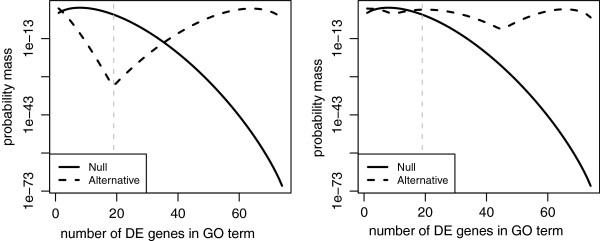
**The conditional probability mass functions given the number of genes in GO:0005524 under a null hypothesis, and alternative hypotheses based on 2-component PMM (left) and 3-component PMM (right).** The grey dashed line is the number of DE genes in GO:0005524.

**Figure 6:**
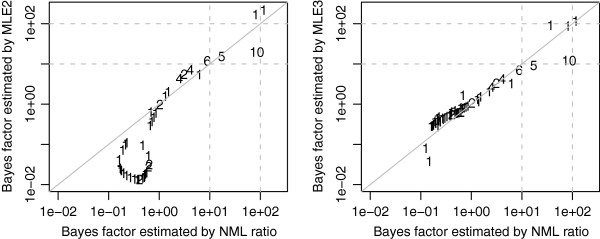
**Comparison of the Bayes factor approximated by the NML ratio with that estimated by MLE2 (left) and MLE3 (right) on the basis of equations (**26**) and (**28**).** The integers are defined in Figure 4. The grey dashed lines mark commonly used thresholds for strong and overwhelming evidence [37,38].

Defining *unrelated* pairs of GO terms as those that do not share any common ancestor, we selected for analysis all unrelated GO molecular function terms with at least 1 DE gene, thereby obtaining a total of 82 GO terms of interest. Figure 4 compares the BBE to the MLEs based on the 2-component (MLE2) and 3-component (MLE3) PMM. Figure 5 displays the probability mass of GO:0005524 under the null and alternative hypotheses of hypothesis comparison (7). Figure 6 compares MLE-based estimates of the Bayes factor given by equation (28) to the NML ratios given by equation (28).

For two GO terms, opposite conclusions would be drawn about their enrichment, depending on which estimator is used. As seen in Figure 4, the estimated LFDRs of GO:0051082 and GO:0005524 using MLE2 were 100%. However, the LFDRs estimated by MLE3 were essentially 0.

Using the MLE formula shown in equation (19), and the *k*-component PMM shown in equation (16), we conclude that the sensitivity of the LFDRs of GO term *i* estimated by MLE2 and MLE3 depended mainly on the sensitivity of the Bayes factor, based on the number of PMM components. Comparing the probability masses of GO:0005524, based on the 2- and 3-component PMMs shown in Figure 5, we found that the probability mass of GO:0005524 under the null hypothesis is larger than that under the alternative hypothesis based on the 2-component PMM (left plot in Figure 5). In contrast, the probability mass under the null hypothesis is smaller than that under the alternative hypothesis based on the 3-component PMM (right plot in Figure 5). Thus, the LFDR estimated by MLE is strongly dependent on the number of PMM components.

While a real data set can in that way indicate the impact of selecting an appropriate method, that impact does not in itself say which method has lowest bias. For that, we rely on the simulation study of the previous subsection.

## Conclusions

As seen in Figure 1 and Figure 2, HBE and HBE-EN have relative high biases for a small number and a medium number of GO terms, respectively. The performance comparison displayed in the left-hand side of Figure 3 indicates that BBE contains the lowest minimum estimated LFDR bias for a small number of GO terms (i.e., 2-32 GO terms) when the proportion of non-enriched GO terms is 1. Although the minimum bias of BEE is not the lowest for some *Π*_0_s under a small number of GO terms, it is very close to the lowest value of bias based on plots shown in Figure 2. The right-hand side of Figure 3 indicates that MLE3 has the lowest maximum absolute estimated LFDR bias in 100 GO terms. MLE exhibits bias similar to that of BBE when the number of GO terms is much larger than *k* except for when the proportion of non-enriched GO terms is high (close to 1). Moreover, MLE3 has lower bias than MLE2 as an LFDR estimator. Due to its conservatism and freedom from PMM, we recommend using BBE for a small number of GO terms of interest (2-32 GO terms) and MLE for a medium number of GO terms of interest (100 GO terms).

Finally, we recommend that NMLE be used when there is only 1 GO term of interest since none of the other estimators is able to estimate LFDR in such a case except by conservatively giving 1 as the estimate. Otherwise, unless *Π*_0_ is known with sufficient accuracy, NMLE is not recommended since it depends not only on the data but also on a guess of the value of *Π*_0_, which in the absence of strong prior information, is often set to the default value of 50%. A closely related approach is to use the logarithm of the NML ratio as a measure of statistical support for the enrichment hypothesis [30] without guessing *Π*_0_. By using 10 and 100 as thresholds of the approximate Bayes factors from equations (26) and (28) to determine whether a GO term is enriched, we reached similar conclusions with both NML and MLE (Figure 6). Thus, in our data set, the NML ratio tends to estimate the Bayes factor almost as accurately as methods that simultaneously use information across GO terms. While we do not expect the same for all data sets, we note that similar results have been found for an application of a modified NML [29] to a proteomics data set [30].

## Competing interests

The authors declare that they have no competing interests.

## Authors’ contributions

ZY implemented the NMLE function, co-designed and executed the simulation study, carried out the comparison among 3 LFDR estimators (BBE, MLE, and NMLE) using the breast cancer data, and drafted the manuscript. ZL implemented the 3-component MLE functions. DRB suggested and guided the project, contributed to writing the paper, co-designed the simulation study, and provided the BBE function. All authors have read and approved the final manuscript.
